# Inputs to Thirst and Drinking during Water Restriction and Rehydration

**DOI:** 10.3390/nu12092554

**Published:** 2020-08-24

**Authors:** Lawrence E. Armstrong, Gabrielle E. W. Giersch, Leslie Dunn, Aidan Fiol, Colleen X. Muñoz, Elaine C. Lee

**Affiliations:** 1 Human Performance Laboratory, Department of Kinesiology, University of Connecticut, Storrs, CT 06269, USA; gabrielle.giersch@uconn.edu (G.E.W.G.); leslie.dunn@pop.belmont.edu (L.D.); aidan.fiol@uconn.edu (A.F.); elaine.c.lee@uconn.edu (E.C.L.); 2 Department of Nutritional Sciences, University of Connecticut, Storrs, CT 06269, USA; 3 Department of Health Sciences, University of Hartford, West Hartford, CT 06117, USA; cmunoz@hartford.edu

**Keywords:** dehydration, osmolality, sensation, drinking, brain, oropharyngeal

## Abstract

Current models of afferent inputs to the brain, which influence body water volume and concentration via thirst and drinking behavior, have not adequately described the interactions of subconscious homeostatic regulatory responses with conscious perceptions. The purpose of this investigation was to observe the interactions of hydration change indices (i.e., plasma osmolality, body mass loss) with perceptual ratings (i.e., thirst, mouth dryness, stomach emptiness) in 18 free-living, healthy adult men (age, 23 ± 3 y; body mass, 80.09 ± 9.69 kg) who participated in a 24-h water restriction period (Days 1–2), a monitored 30-min oral rehydration session (REHY, Day 2), and a 24-h ad libitum rehydration period (Days 2–3) while conducting usual daily activities. Laboratory and field measurements spanned three mornings and included subjective perceptions (visual analog scale ratings, VAS), water intake, dietary intake, and hydration biomarkers associated with dehydration and rehydration. Results indicated that total water intake was 0.31 L/24 h on Day 1 versus 2.60 L/24 h on Day 2 (of which 1.46 L/30 min was consumed during REHY). The increase of plasma osmolality on Day 1 (297 ± 4 to 299 ± 5 mOsm/kg) concurrent with a body mass loss of 1.67 kg (2.12%) paralleled increasing VAS ratings of thirst, desire for water, and mouth dryness but not stomach emptiness. Interestingly, plasma osmolality dissociated from all perceptual ratings on Day 3, suggesting that morning thirst was predominantly non-osmotic (i.e., perceptual). These findings clarified the complex, dynamic interactions of subconscious regulatory responses with conscious perceptions during dehydration, rehydration, and reestablished euhydration.

## 1. Introduction

As essential aspects of optimal physiological function and human survival, intracellular and extracellular fluid concentrations and total body water are regulated by a complex, dynamic network of sensory nerves, autonomic neuroendocrine responses, and central integration at specific brain loci [1,2,3]. Intracellular dehydration, movement of extracellular water into cells, and the resulting increase of plasma osmolality which is detected by central osmosensors, modulate thirst, drinking behavior, and renal water retention (i.e., via the antidiuretic hormone arginine vasopressin) to stabilize the volume and concentration of the extracellular fluid [4,5]. Extracellular hypovolemia results in decreased blood volume and arterial pressure, which in turn stimulate vascular receptors that signal the brain to modulate thirst, drinking, and renal sodium retention via the hormone angiotensin II [2]. Thus, perceived thirst is centrally integrated with subconscious autonomic neuroendocrine responses [6,7,8] to maintain a narrow physiological range of body fluid osmolality, volume, and blood pressure [9]. In addition to thirst, other conscious perceptions influence drinking behavior and the control of vasopressin; originating as neural signals from the oropharyngeal region and gut [10,11], they are perceived as mouth dryness or wetness and stomach emptiness or distention [12,13].

Thirst and drinking behavior apparently are predominantly influenced by subconscious autonomic responses in some situations and by conscious perceptions and sensations at other times [4,9,14]. However, the following aspects of afferent inputs to the brain have not been well defined: the hourly time course, relative intensity, and duration/interactions of perceived sensations (e.g., thirst, mouth dryness, stomach emptiness) which may either reinforce or counteract inputs that signal hydration status (e.g., plasma osmolality, body water deficit) during prolonged dehydration. Further, little is known about the durations or magnitude of change of these afferent inputs to the brain during/after rehydration. Thus, we aimed to examine the dynamic, complex interactions of multiple factors during a 24-h water restriction period, a 30-min monitored rehydration session, and during the reestablishment of euhydration via unrestricted drinking across 24 h. The present investigation assessed subconscious autonomic responses to changing hydration status, conscious perceptions including thirst, and drinking behavior in free-living, healthy adult men, while they conducted usual daily activities. Free-living adults were observed because few previous thirst and drinking behavior studies have involved free-living humans [9]. Measurements assessed body fluid volume/concentration, dietary intake, renal responses, drinking behavior, and ratings of subjective perceptions. In this way, the present investigation provides important context regarding the central integration of information, as identified in animal models and previously published human brain imaging studies [12].

## 2. Materials and Methods

### 2.1. Research Design

The University of Connecticut Institutional Review Board for Human Studies approved this research (Protocol no. H17-291, approved 30 January 2018), and each subject provided written informed consent to participate during an informational meeting that described the purposes, risks, benefits, time commitment, and procedures of participation. Each person completed a medical history questionnaire. This history was examined by a physician who verified that participants had no apparent illness and met none of the following exclusionary criteria: kidney disease or a chronic illness that altered fluid–electrolyte balance; medications that affected kidney function (e.g., diuretics or blood pressure medications); use of a tobacco product; or chronic illness including diabetes. Participants were instructed to abstain from exercise and alcohol consumption on all days, and to retire for sleep each night as they do during typical weeknights.

Test subject selection involved convenience sampling. Participants were recruited from the university campus and surrounding community via e-mail and internet announcements, print media, bulletin boards posters, and verbal announcements at group meetings. Because previous research experiences in this community had shown that adults aged 21–35 years were optimally compliant with experimental instructions (e.g., no alcohol intake, no exercise), and because the present research protocol required great cooperation and attention to detail (e.g., hourly data recording and multiple laboratory visits across consecutive days), the subject sample was limited to this age group. Screening and enrollment were open to men from all ethnic groups, income groups, and education levels. Women were not included in this investigation (i.e., which involved dehydration and rehydration) for the following reasons. First, women experience a natural monthly rhythm of body water change, thus it is optimal to observe women at the same point in the menstrual cycle (e.g., mid-luteal phase). Laboratory verification of the menstrual phase (e.g., luteinizing hormone surge) is time-consuming and costly. Second, contraceptive use and contraceptive type represent potential confounding effects on body water volume and tonicity. Third, coordinating test subject schedules with menstrual phase and laboratory availability places women at a disadvantage versus men in terms of longer duration of participation, additional testing requirements, and greater inconveniences.

This investigation was part of a large project that will result in multiple manuscripts, each with unique research questions regarding dehydration. To avoid duplication of published data, all tables and figures in the present investigation are original.

**Figure 1:**
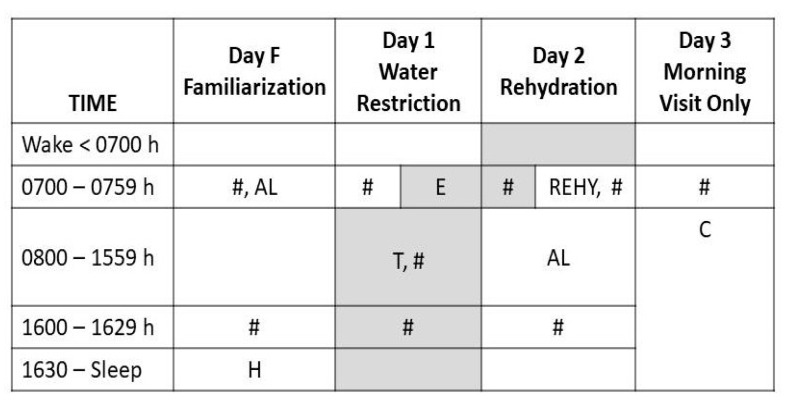
Perceptual ratings were recorded by test participants at the beginning of every waking hour. All ingested food and fluid was recorded in a diary. A single small urine sample was collected upon waking each morning; all other urine was collected in a 24-h container. Abbreviations: #, visited laboratory for body weight, blood and urine samples; AL, consumed water and food ad libidum for 24 h; H, evening hydration procedure (500 mL water consumed above ad libidum intake); E, experimental intervention began (gray shaded zone; ~0730 h on Day 2 to ~0730 h on Day 3), subjects drank no fluids and ate dry foods; T, when thirst was first sensed, the subject notified investigators and returned to the laboratory; REHY, consumed water ad libidum during 30-min seated laboratory observation period; C, conclusion of research participation.

Prior to the beginning of testing, subjects met once with investigators to receive instructions regarding record keeping, food diaries, perceptual ratings, sample collections, and the study timeline as depicted in Figure 1. Test subjects were instructed to conduct daily activities (e.g., work, meals, attending work or university classes) as they typically would. Measurements, sample collections and observations were recorded across 48 h, during visits (Monday through Friday, 20–60 min per visit) to the Human Performance Laboratory (HPL). During each visit to the HPL, participants provided a small urine sample, rated perceptions and sensations as described below, and had a small blood sample drawn from an antecubital vein. Morning HPL visits (0700–0759 h, see Figure 1) occurred after the first-morning bladder void and before eating breakfast or drinking fluid (i.e., in a 9–13 h fasted state); afternoon visits occurred between 1600–1629 h.

The first day of participation (Day F) was designed to familiarize test participants with the daily schedule, procedures, and measurements. During the morning HPL visit, measurements (i.e., body weight, height, age, perceptual/mood ratings, blood sample and urine sample) were recorded by investigators. Test participants consumed their usual diet ad libitum throughout Day F. On the evening of Day F, they consumed 500 mL of water in addition to the water they had consumed earlier in the day, to increase the likelihood that they would arrive at the HPL in a euhydrated state on the morning of Day 1 (baseline).

All consumed food and fluid items were recorded in a food diary, including number, volume, size, brand, manufacturer, and method of preparation; nutrition labels and food packages were submitted when available. Because dietary analyses utilized 24-h diet records, macronutrient components were analyzed (Nutritionist Pro™ Diet Analysis Software, Axxya Systems, Redmond, WA, USA) to report descriptive statistics for the water restriction intervention (Day 1) and ad libitum rehydration (Day 2).

Subjects provided single urine samples (~200 mL) in clean, inert sample cups, upon waking each morning and during each visit to the HPL. They collected all other urine in clean, inert containers which were provided by investigators and were carried in a cloth bag during all daily activities. The volume of each single urine sample that was not analyzed immediately was poured into the 24-h collection container.

### 2.2. Experimental Intervention

Beginning in a euhydrated state on the morning of Day 1, all test participants consumed no fluid and ate dry foods containing low water content for 24 h. This experimental intervention occurred between the morning HPL visits on Day 1 and Day 2, and is illustrated in Figure 1 as a shaded gray zone. This eating plan was guided by a list of acceptable dry foods (e.g., bread, granola bars, nuts, dry cereal) and unacceptable watery foods (e.g., soup, watermelon, oranges, grapes, smoothies, and milk). On Day 1, test subjects remained on the university campus and, when they first became aware of thirst during daily activities, they contacted an investigator via mobile phone then returned to the HPL to have bodyweight measured, provide a venous blood sample and a small urine sample, and to record perceptual/mood ratings. This point was defined as “thirst awareness”.

The water restriction phase of testing ended during the 60-min morning HPL visit on Day 2. Following morning measurements, each subject sat at a table with an investigator and rehydrated by consuming water ad libitum for 30 min (REHY). The volume of ingested water was measured (benchtop pan balance, ±1 g) then morning measurements were repeated. When leaving the HPL to conduct daily activities, subjects received one 600 mL bottle of flavored electrolyte replacement beverage plus three 500 mL bottles of mineral water which they carried during daily activities, and were encouraged to drink ad libitum during the subsequent 24 h.

### 2.3. Laboratory Measurements and Analyses

Calibrated digital floor scales (Ohaus Inc., Florham Park, NJ, USA, model DS44L) measured body weight with a precision of ±100 g, during each HPL visit. The 24-h volume of urine was measured gravimetrically (±0.1 g) on a laboratory benchtop scale (Ohaus Inc., Parsippany, NJ, USA). Urine specific gravity was assessed with a hand-held refractometer (Atago Co., Tokyo, Japan, model 300 CL).

During each visit to the HPL, one blood sample was drawn from an antecubital vein and was centrifuged at 3000 rpm for 15 min. The resulting plasma samples were frozen at −80 °C. Urine and plasma sample osmolalities were measured in duplicate (200 μL each) with a freezing-point depression osmometer (Advanced Instruments, Inc., Norwood, MA, USA; model OsmoPRO).

### 2.4. Visual Analog Scales (VAS)

Ratings of perceptions and sensations were self-reported by participants during each HPL visit and throughout all days. VAS consisted of a question or statement and a 100 mm straight line with left and right bipolar anchors (adjectives or descriptive phrases). One end of the line represented the low rating/intensity of the variable and the other end the high rating/intensity. Subjects recorded their responses, representing that moment in time, by marking a single line on each VAS scale. The distance along the 100 mm line (i.e., measured from the low rating/intensity of the variable) represented the subject’s rating. The following four items and bipolar anchors were presented in all VAS packets: How thirsty are you? (not at all—very); I desire water ____ (very little—very much); My stomach feels ____ (very full—very empty); My mouth feels ____ (very moist—very dry). Twelve other VAS items and bipolar anchors were included in each stapled packet but those data (unpublished) will be described in a future manuscript. Each VAS appeared on a separate piece of paper (5 cm × 20 cm), in a small stapled packet containing the date and hour. On each occasion, a different blank packet of VAS was used. The order of VAS questions was randomized, to reduce the likelihood of an order effect. The positions of VAS bipolar anchors (e.g., left versus right) also were randomized across the packets. These design features were based on the methods described by Rolls et al. [15] and Phillips et al. [16].

### 2.5. Statistical Analyses and Figure Construction

Data are reported as means ± standard deviation (SD). A repeated-measures analysis of variance tested the differences among group means (e.g., change across time) in the present sample (Excel ver. 14.0, Microsoft Corp., Redmond, WA, USA). If a significant main effect was identified, post hoc comparisons were conducted using Student’s *t*-tests with Bonferroni corrections based on the number of comparisons. An alpha level of 0.05 was used for all significance tests. The number of data points varies across variables and time points (n = 14–18) due to small or no blood samples during phlebotomy, test subject inability/forgetting to record some hourly VAS ratings, and test subject inability to attend all HPL visits at the prescribed time. Figures were created with commercial software (KaleidaGraph, ver. 3.5×, Synergy Software Inc., Reading, PA, USA).

### 2.6. Calculation of Sample Size

This investigation was part of a multi-investigator project that included multiple research questions regarding dehydration. The sample size calculation was based on the variability of body mass loss, which represented the level of dehydration experienced by each participant. Considering previous studies conducted in our laboratory (i.e., in which the standard deviation of body mass loss was 0.8% and the day-to-day between-subject variation was approximately 50%), we anticipated that the smallest change in body mass would be 1.5%. We selected an effect size of 0.2 and the maximum chance of a Type 1 error was set at 5% (very unlikely). For the present experimental design, which included a pre- and post-dehydration weight measurement for each subject, the minimal sample size to detect a significant difference (*p* < 0.05) was computed as n = 12. The present participant sample (n = 18) exceeded this minimum size.

## 3. Results

The 18 men who participated in this investigation were members of the university community and had the following personal characteristics (mean ± SD): age, 23 ± 3 y; body mass, 80.09 ± 9.69 kg; height, 176 ± 6 cm. During the evening of Day F, participants were instructed to consume 500 mL of water above their typical ad libitum daily fluid intake; they also were instructed to avoid exercise and alcohol intake throughout testing. These procedures successfully achieved stability of body water and extracellular concentration on the morning of Day 1. The following measurements were recorded by investigators during HPL visits on the afternoon of Day F (1600 h) and the morning of Day 1 (0700 h), respectively: body mass, 80.29 ± 10.60 vs. 80.11 ± 10.58 kg (representing a difference of 0.22 ± 0.61%); single sample urine specific gravity, 1.017 ± 0.009 vs. 1.020 ± 0.007; plasma osmolality, 296 ± 6 vs. 296 ± 4. Thus, we considered test participants to be euhydrated on the morning of Day 1, at the beginning of water restriction.

Table 1 presents dietary macronutrient intakes on Day 1 (experimental intervention) during which no fluids and only dry food items were ingested, and Day 2 (rehydration) during which water, beverages and food items were consumed ad libitum. The Day 1 and Day 2 mean values were compared via paired, two-sample *t*-tests with Bonferroni corrections. The food moisture content (row 2 in Table 1) provides evidence of test subject compliance with the dietary intervention, in that participants consumed an average of only 0.31 L of food moisture on Day 1 versus 1.13 L on Day 2 (*p* = 0.0002); this resulted in mean total water intakes of 0.31 and 2.60 L/24 h on Days 1 and 2, respectively (*p* = 3.2 × 10^−8^). The other dietary components in Table 1 were statistically similar between days. Table 2 also illustrates successful test subject compliance with the 24-h water restriction and rehydration protocols in that all hydration indices were significantly different (*p* = 0.003 to 1.7 × 10^−10^) on Days 1 and 2.

### 3.1. Perceptual Ratings Were Strongly Correlated to Thirst

**Figure 2:**
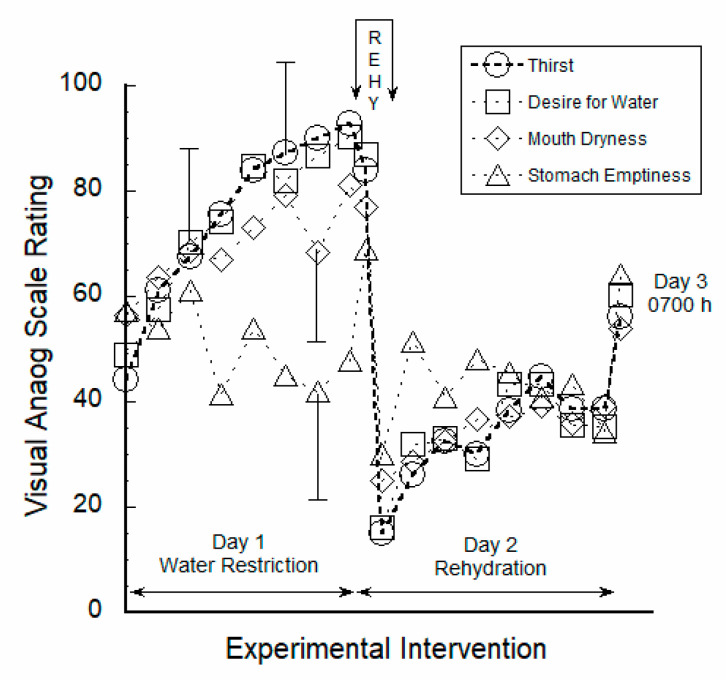
Visual Analogue Scale (VAS) ratings for thirst, desire for water, mouth dryness, and stomach emptiness during water restriction and rehydration. Values are means (n = 14–18) and representative error bars indicate the median standard deviation of each variable. The REHY zone (top center) represents a 30-min monitored water consumption session. The mean VAS ratings measured during the thirst awareness visit to the laboratory (Day 1) are not depicted in this figure but appear in Table 3, column 3.

Participants reported their perceptions by marking VAS during each HPL visit and throughout all days of testing, as described in the Methods section above. Figure 2 illustrates the trends of VAS ratings (i.e., each with a scale of 0–100) for the following four questions/statements: How thirsty are you? I desire water ____, My mouth feels ____, and My stomach feels ____. Thus the top of the *y*-axis in Figure 2, with a rating of 100, represents being very thirsty, desiring water very much, feeling a very empty stomach, and feeling a very dry mouth. The 24-h water restriction phase began at 0730 h of Day 1 and ended at 0730 h of Day 2. The mean ± SD values for these four VAS ratings (94 data points, Days 1–3) were: thirst, 63 ± 30; desire for water, 64 ± 30; mouth dryness, 40 ± 27; stomach emptiness, 51 ± 22. The 24-h rehydration phase began at 0730 h of Day 2 with a seated 30-min unlimited access ad libitum rehydration session, and ended at 0730 h of Day 3 when subjects completed their participation in this study.

Thirst VAS ratings were correlated via linear regression with the other variables in Figure 2 (18 subjects, 164 data points across 3 days), with the following results: thirst vs. desire for water, r2 = 0.73, *p* = 1.0 × 10^−48^; thirst vs. mouth dryness, r2 = 0.45, *p* = 3.9 × 10^−23^; thirst vs. stomach emptiness, r2 = 0.05, *p* = 0.005. Thus, mouth dryness and stomach emptiness independently accounted for 45% and 5%, respectively, of the variance in thirst. Further, the strong correlation between VAS ratings of thirst and desire for water (r2 = 0.73), and the close proximity of these two variables at all time points in Figure 2 (i.e., see circle and square symbols), indicate very strong internal validity.

### 3.2. Thirst Correlated with Hydration Indices during Water Restriction

During water restriction, thirst VAS ratings also were correlated via linear regression with plasma osmolality (86 data points across six HPL visits) and body mass change (70 data points across six HPL visits); the following results were obtained: thirst vs. body mass change (kg), r2 = 0.24, *p* = 0.000014; thirst vs. body mass change (%), r2 = 0.24, *p* =0.000014; thirst vs. plasma osmolality, r2 = 0.09, *p* = 0.0046; thirst vs. plasma osmolality change, r2 = 0.05, *p* = 0.035. Thus, body mass and plasma osmolality independently accounted for 24% and 5–9%, respectively, of the variance in thirst ratings.

**Figure 3:**
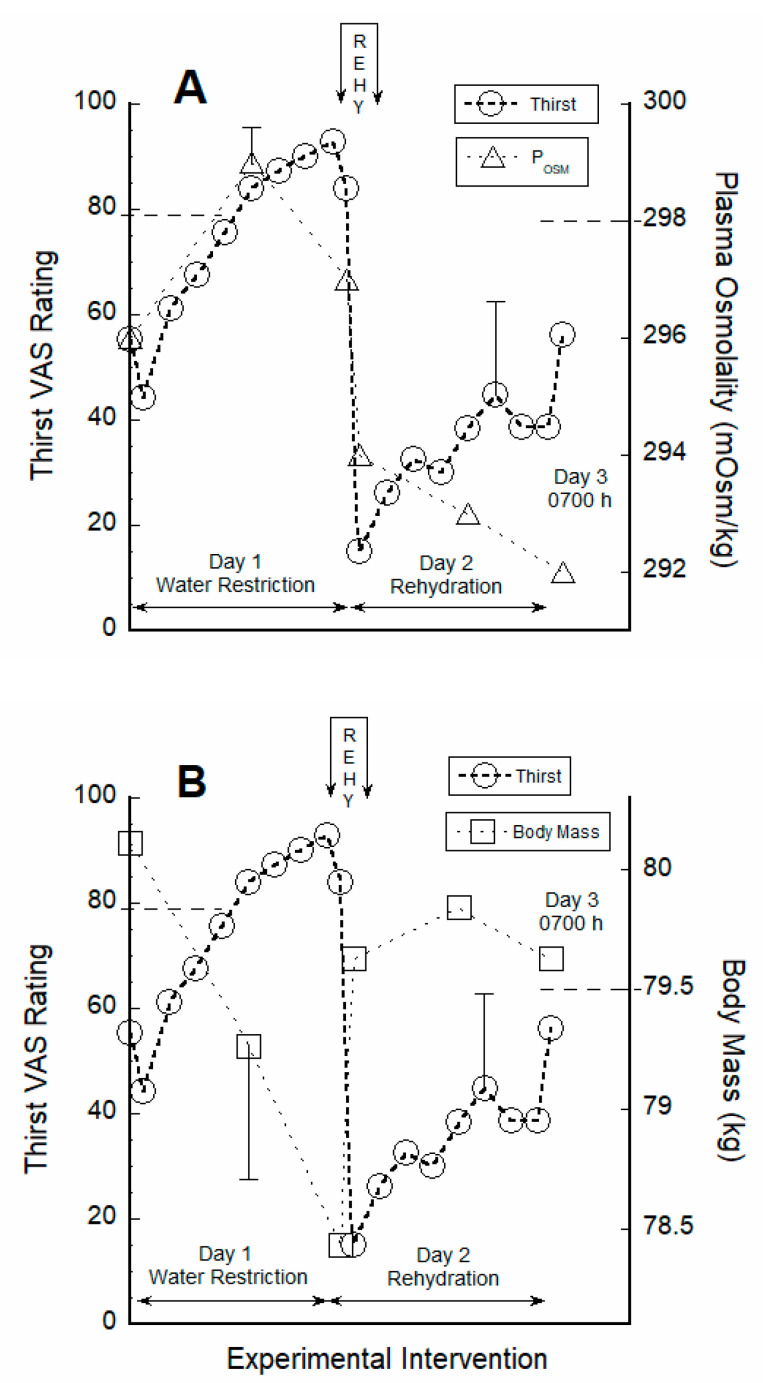
Relationships between visual analogue scale (VAS) ratings of thirst and plasma osmolality (P_OSM_; panel **A**) and between thirst and body mass (panel **B**). The dual y-axes are scaled to span the approximate minimum and maximum values of each variable. The dissociation of plasma osmolality and thirst appeared only on Table 3. (lower right quadrant of panel **A**). The re-emergence of euhydration is represented by body mass values on Day 3 (upper right quadrant of panel **B**) that are statistically similar to baseline (initial value on Day 1). The horizontal dashed lines that intersect each axis represent the group mean values measured during the thirst awareness visit to the laboratory on Day 1.

Figure 3 depicts the temporal relationship of thirst ratings (open circles) to plasma osmolality (panel A) and to body mass (panel B) during the 24-h water restriction phase and the 24-h rehydration phase. The dual y-axes in these graphs are scaled to span the approximate minimum and maximum values of each variable. The horizontal dashed lines in panels A and B represent the mean value on Day 1 when test subjects became aware of thirst and returned to the HPL. Table 3 describes measurements recorded during this thirst awareness visit to the HPL, which occurred at a different time for each participant.

### 3.3. VAS Ratings Responded to 30-Min Fluid Intake

Figure 2 and Figure 3 illustrate the 30-min rehydration session, during the Day 2 morning visit to the HPL, as a zone labeled REHY (top center). Subjects did not consume food or fluids before arriving at the HPL, but resumed ad libitum eating and drinking after leaving the laboratory. The rapid decrease of all VAS ratings occurred during REHY due to consumption of 1.41 ± 0.40 L (17.8 ± 4.4 mL/kg body mass) of water in 30 min. Before REHY, during 24 h of water restriction, the mean ± SD VAS values (n = 66 data points) were: thirst, 77 ± 18; desire for water, 77 ± 20; mouth dryness, 71 ± 21; and stomach emptiness, 55 ± 21. Across the 12 h after REHY, the mean VAS values (n = 60 data points) were: thirst, 34 ± 23; desire for water, 37 ± 24; mouth dryness, 36 ± 20; and stomach emptiness, 44 ± 23. Between 0730 h and 1200 h on Day 2, thirst and desire for water remained low, mouthfeel was moist, but the mean stomach sensation evolved from being full to a neutral midpoint between 0800 h and 1000 h. We interpret this latter effect as the result of gastric emptying of ingested water. Similarly, Figure 2 shows that the sensation of stomach emptiness (triangle-shaped symbols) was cyclic, roughly coinciding with the hours of meal consumption. Mouth dryness (diamond-shaped symbols in Figure 2) also vacillated, but less distinctly than stomach emptiness. Mouth sensations likely were influenced by the dry food items eaten during water restriction on Day 1 (see food moisture in Table 1).

### 3.4. Prior Hydration Status and VAS Ratings Did Not Predict REHY Volume

To examine seven factors that may have influenced the amount of water consumed ad libitum at the end water restriction on Day 2 during REHY, this 30-min intake volume was correlated via linear regression with four VAS ratings on Day 2 at 0700 h (thirst, desire for water, mouth dryness, stomach emptiness; n = 16) and three participant characteristics (height, body mass, BMI; n = 17). No personal characteristic or VAS rating was significantly correlated at *p* < 0.05 with the volume of water consumed during REHY. Among these 14 comparisons (analyzed as L/30 min and mL/kg/30 min), the coefficients of determination (r2) ranged from 0.003 to 0.21, and P values ranged from 0.06 to 0.95. Similarly, to examine the ways that the 24-h water restriction protocol (Day 1) may have influenced the amount of water consumed on Day 2 during REHY, the 30-min intake volume was correlated via linear regression with plasma osmolality change (from Day 1 at 0730 h to Day 2 at 0730 h), single plasma osmolality measurement prior to REHY (Day 2 at 0730 h), and body mass change (from Day 1 at 0730 h to Day 2 at 0730 h). None of these three variables was significantly correlated at *p* < 0.05 with the volume of water consumed (analyzed as L/30 min and mL/kg/30 min) during REHY. Among these six comparisons, the coefficients of determination (r2) ranged from 0.001 to 0.07, and *p* values ranged from 0.40 to 0.95. In the Discussion section below, we propose three explanations as to why none of these 20 variables predicted the 30-min volume consumed during REHY. Hindering complete explanations is our current inadequate understanding of the brain’s conversion of hydration-relevant homeostatic signals and subjective perceptions into motivation and drinking behavior.

## 4. Discussion

Several regulated factors, each with a distinctive homeostatic brain set point (i.e., defined here as a narrowly-encoded range that optimizes bodily functions), are known to induce or inhibit thirst, including blood concentration, volume, pressure, and hormone concentrations [5,9,17]. However, many aspects of human drinking behavior cannot be explained by classic homeostatic models [8]. The consideration of conscious sensations (e.g., dry mouth, stomach distention) [15], and subconscious oropharyngeal or gastrointestinal afferent nerve signals which are stimulated by drinking, swallowing, and the presence of water or salt in the gut [13,17], provides a more thorough paradigm of thirst and satiation.

The present investigation described the interactions of physiological and perceptual factors during the emergence of thirst due to dehydration (Day 1), the rapid decay of thirst during rehydration (Day 2, 30-min REHY), and the presence of mild thirst despite reestablished euhydration (Day 3, 0700 h). Table 1 provides evidence that test participants complied with the experimental intervention on Day 1 by consuming no fluids, and a total water intake of only 0.31 L/24 h provided by moisture in solid foods. This volume is considerably less than the Adequate Intakes for water recommended for men by the European Food Safety Authority (2.5 L·d^−1^ [18]) and the U.S. National Academy of Medicine (3.7 L·d^−1^ [19]). Table 2 describes hydration indices that indicate test subject compliance with the water restriction and dry food protocol. When compared to previously published categories of hydration status, determined for healthy young men conducting daily activities with unrestricted food and water intake [20], the Day 1 hydration indices (Table 2, column 2) represent “very dehydrated” (i.e., single morning sample urine specific gravity, 24-h urine specific gravity, 24-h urine osmolality) and “extremely dehydrated” (i.e., 24-h urine volume, morning serum osmolality) states. Thus, the present experimental design successfully introduced progressive mild dehydration (−2.12 ± 0.50% of body mass) across 24 h, and allowed us to observe the development of thirst during daily activities.

### 4.1. Responses to Water Restriction

As a result of the combined effects of low water intake, 4.4 g sodium (Table 1), and intestinal absorption of dry food items which added osmolytes to the extracellular fluid and increased blood concentration, plasma osmolality increased on Day 1 from 0700 h through 1600 h. An increase in the extracellular concentration of non-permeable osmolytes causes an osmotic movement of water out of body cells (i.e., intracellular dehydration), cerebral osmoreceptors sense this concentration change, and thirst is stimulated [10,17]. Subsequently, mean plasma osmolality (Figure 3, panel A) decreased slightly from the afternoon of Day 1 (299 ± 7 mOsm/kg at 1600 h) to the morning of Day 2 (297 ± 5 mOsm/kg at 0700 h), including the overnight hours. We postulate that this decrease occurred as the result of intracellular water moving into the circulation, as well as renal regulation of water and osmolyte excretion (Table 2).

There is practical value in a plasma osmolality threshold for thirst that appears before water-seeking behavior. If this threshold did not exist, cognitive attention would be aroused by minor deviations from baseline, and other physiologically important behaviors would be minimized by a constant preoccupation with thirst [21]. In fact, multiple controlled laboratory studies have determined the plasma osmolality at which human thirst is perceived [22,23], but a precise universal threshold cannot be generalized to all individuals because it varies by sex (men, women), menstrual cycle phases (follicular, luteal), and twin types (monozygotic, dizygotic [12]). Indeed, when test subjects left their daily activities on Day 1 and returned to the HPL because they had become aware of thirst (Table 3), mean plasma osmolality had risen from the baseline value of 296 ± 4 to 298 ± 4 mOsm/kg, indicating that the plasma osmolality threshold for the onset of thirst had been reached. This was, to our knowledge, the first time that the plasma osmolality threshold for the onset of thirst was determined in free-living adults. Two previous publications reported similar mean thirst thresholds of 297–298 mOsm/kg for men and women [22,23] during controlled laboratory intravenous infusions of hypertonic saline. Further, Day 1 data suggest that plasma osmolality signals during water restriction (Figure 3, panel A) were centrally integrated with, or were potentiated by, concurrent perceptions of thirst and desire for water (Figure 2).

As depicted in Figure 2, the mean VAS ratings of thirst and desire for water at 0700 h of Day 1 represented a neutral, euhydrated state (thirst, 55 ± 14; desire for water, 55 ± 18) but progressively increased in intensity until Day 2 at the end of water restriction (thirst, 88 ± 14; desire for water, 87 ± 20). The VAS ratings of mouth dryness (Figure 2, diamond-shaped symbols) tracked thirst and desire for water during most of Day 1 water restriction and Day 2 rehydration. This suggests that the sensation of mouth dryness reinforced osmotically-stimulated thirst, or was an important input to thirst, during the present investigation.

Although gastric distention and gastrointestinal contents (e.g., sodium) have been proposed as signals to thirst [3,24,25,26], little is known about the role of the stomach or intestinal sensations in human thirst and drinking behavior. Figure 2 shows that stomach sensations (triangle-shaped symbols) on Days 1 and 2 oscillated, roughly coinciding with the hours of meal consumption and subsequent gastric emptying. Further, the two greatest increases of stomach emptiness occurred overnight during a period of approximately 8–9 h that included sleep, and regression analysis indicated that stomach sensations explained only 5% of the variance in thirst (see Results section). Thus, in contrast to animal research [24,25,26], perceived stomach sensations apparently did not reinforce the perception of thirst in humans during the present investigation. However, these conscious stomach sensations are distinct from subconscious autonomic nervous activities, which are known to influence thirst in rodents. Sophisticated experiments using a mouse model of thirst [3] have demonstrated that the water and salt contents of the gut (i.e., not stomach distention) are precisely measured and communicated to the subfornical organ, one of three main nuclei within the lamina terminalis. This forebrain region integrates gastrointestinal osmolality, oropharyngeal, and bloodborne signals to control the termination of drinking. Observably, the gastrointestinal osmotic state specifies whether the rapid inhibition of thirst neurons by oropharyngeal signals during drinking will be transient or persistent [3]; this neural activity anticipates the need to seek and consume water in the near future by modulating the sensation of thirst. The present data support this mechanism in humans. When dry food and no fluids were ingested for 24 h on Day 1 (Figure 1), the absorption of dietary substances likely resulted in elevated gastrointestinal osmolality and increased plasma osmolality (Figure 3, panel A). Because no fluids were consumed on Day 1, no drinking-induced oropharyngeal signals were generated. Throughout water restriction, VAS ratings increased and peaked during the final self-assessment on Day 1 (Figure 2). This scenario is consistent with the concept of afferent signals (i.e., gastrointestinal, bloodborne, and oropharyngeal) and perceived mouth dryness working in concert to motivate persistent drinking.

### 4.2. Acute 30-Min Rehydration (REHY)

The 24-h water restriction phase of this investigation ended when subjects drank ad libitum with unlimited access to water for 30 min; this session allowed us to observe drinking behavior. The average volume consumed during this period was 1.41 ± 0.40 L/30 min (17.8 ± 4.4 mL/kg body mass/30 min). This acute water intake rapidly extinguished thirst, the desire for water, mouth dryness, and stomach emptiness; all VAS ratings decreased to their lowest point during the entire investigation (Figure 2). A similar human study reported that thirst-relevant VAS ratings decreased to their nadir in less than 10 min after drinking began [15].

Although we did not record the volume consumed at intervals during REHY, Rolls and colleagues previously described the drinking behavior and hematological constituents of healthy young men, following a 24 h water restriction period [15]. Their relevant findings were as follows: (a) 65% of the total 60-min water intake was consumed during the first 2.5 min of access to water; (b) after 2.5 min, drinking proceeded relatively slowly and the volume of each intake decreased rapidly; and (c) the mean volume consumed during the initial 2.5 min was 372 mL, whereas the volume of all intakes after 2.5 min averaged only 52 mL. On the basis of plasma sodium and osmolality, Rolls and colleagues concluded that the movement of ingested water into cells could not account for the early and rapid decrease in the rate of drinking that occurred within the initial 2.5 min of drinking. Instead, they emphasized the importance of VAS ratings (i.e., similar to those depicted in Figure 2) during the first few minutes of access to water [15]. Table 3 supports this observation in that body mass and plasma osmolality had not changed significantly at the point of thirst awareness, whereas three VAS ratings increased significantly.

**Figure 4:**
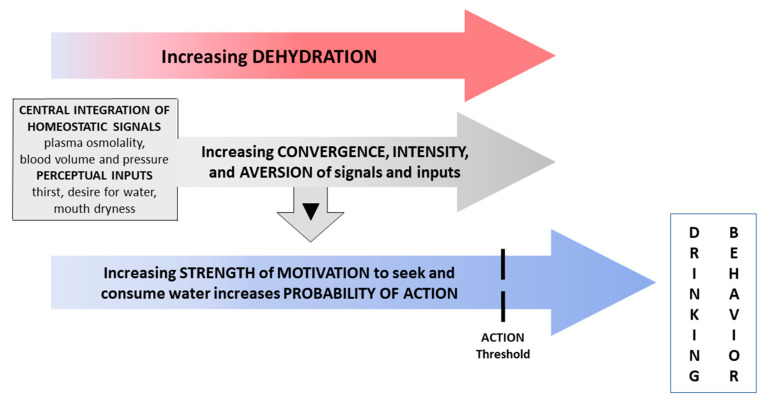
In the present investigation, the existence and intensity of physiological signals and perceptual inputs vary, depending on hydration status. We propose that, with increasing dehydration in humans, the convergence of physiological signals and perceptual inputs, their intensity, and their increasing aversive mood create increased strength of motivation to seek water and drink. Our current insufficient understanding of the conversion of subconscious homeostatic signals and conscious subjective perceptions into human motivation and behaviors (▼symbol) relies on few recent animal studies.

In the present investigation, none of the following variables was significantly correlated with the volume of water consumed during REHY (Day 2, 30 min duration): height, body mass, body mass index; change of body mass and plasma osmolality during water restriction; plasma osmolality immediately prior to REHY; VAS rating of thirst, desire for water, mouth dryness, and stomach emptiness immediately prior to REHY. Although our current understanding of the brain’s conversion of subconscious homeostatic signals and conscious subjective perceptions into motivation and drinking behavior is inadequate (Figure 4), we propose three possible explanations as to why the volume of water consumed in a hypohydrated, relatively thirsty state was not correlated with thirst, other VAS ratings, or homeostatic variables (i.e., plasma osmolality, body mass change). (1) Drinking behavior is the result of a diverse neural network that is not identical to the one that potentiates and extinguishes thirst [3,12,27]. Indeed, indescribable complexity was the exact conclusion drawn from an assessment of primate water intake that was electrically stimulated at 5885 forebrain loci. Neither response probability, response distribution and threshold, nor behavioral analysis provided a simple anatomical or behavioral scheme that satisfactorily explained drinking behavior [28]. (2) Other biological variables, (e.g., intestinal water content and concentration [3]) not measured during the present investigation, may influence thirst and/or drinking behavior. (3) One or more factors may be strongly correlated with thirst and the desire for water at specific times but not at others. Figure 2 and Figure 3 illustrate this concept. During the initial 8 h of water restriction, all signals to thirst and the desire for water rose in unison (Figure 2), including a rise of plasma osmolality (Figure 3) beyond the central set point for thirst onset (see above); strong statistical correlations would be expected during this phase (i.e., when signals act synchronously). In contrast, during REHY and ad libitum drinking on Day 2, the patterns of change for plasma osmolality and VAS ratings differed and their synchrony decreased, in part because plasma osmolality fell below the central set-point for thirst [22,23]; weak, non-significant statistical correlations would be expected during this phase.

### 4.3. Re-Emergence of Body Water Homeostasis and Mild Thirst

The baseline mean body mass (80.11 ± 10.58 kg on Day 1 at 0700 h) decreased by 1.67 ± 0.35 kg (2.12 ± 0.50%) during 24 h of water restriction. Immediately after the 30-min REHY session on Day 2, body water was replenished to the point that the mean body mass was 79.63 ± 10.79 kg and remained stable across the subsequent 23 h (Day 2 at 1600 h, 79.84 ± 10.87 kg; Day 3 at 0700 h, 79.63 ± 10.48 kg), despite a urine output of 1.77 L/24 h on Day 2 (Table 2). Using a published linear regression equation that incorporated male age, height and body mass [29], we calculated the test subject mean total body water as 45.99 L. Thus, the difference between the Day 1 morning euhydrated baseline and the Day 3 morning measurement was ~1.0% of total body water and 0.6% of body mass (i.e., a difference of 0.48 ± 0.54 kg). We conclude, therefore, that body water homeostasis was regained in the present investigation by Day 2 at approximately 1600 h (i.e., 8 h after rehydration began) and continued until the end of testing on Day 3 (Figure 3, panels A and B; Table 2, column 4).

Within 90 min of the end of REHY on Day 2, VAS ratings of thirst and the desire for water increased slowly until 1800 h, then plateaued (Figure 2); ratings of mouth dryness followed a similar trajectory. The intensity of these VAS ratings was slightly less than those recorded at the euhydrated baseline on Day 1. It was not until the morning of Day 3 that all VAS ratings exceeded baseline values, suggesting that mild thirst had re-emerged during the final HPL visit. At 0700 h on Day 3, the mean plasma osmolality dissociated from VAS ratings (Figure 2 and Figure 3, panel A), as evidenced by the decreasing trend of plasma osmolality (i.e., due to ongoing water consumption) and the increasing trend of all VAS ratings. This dissociation of physiological signals and perceptual inputs to thirst on the morning of Day 3, which was not evident during water restriction, suggests that (a) increased overnight thirst (i.e., when the average VAS thirst rating rose from 39 to 56; Figure 2) was not due to an increase of plasma osmolality, and (b) early morning perceptions (e.g., mouth dryness and stomach emptiness) predominated over osmotic signals when test subjects were euhydrated. Based on principles derived from animal models, this dissociation phenomenon also suggests that plasma osmolality and conscious perceptions are processed at different brain loci [1,3].

The ability to measure human subjective perceptions and sensations during fMRI imaging offers an advantage over animal experiments, because thirst ratings (Figure 2) can be correlated in real time with activations of specific brain loci. Yet, animal models provide unique insights. For example, one noteworthy project [27] employed an array of implanted microelectrodes to observe 23,881 individual neuron activations at 34 forebrain and midbrain loci, during several hundred thirst-related tasks (e.g., water as a reward for correct choices) performed by dehydrated mice. Data analysis revealed a global representation of the thirst motivational state which modulated the brain-wide propagation of sensory information and its subsequent transformation into drinking behavior. It is reasonable that analogous thirst- and drinking-relevant neural networks, and central integrative processes (i.e., perhaps more complex), exist in the human brain (Figure 4). We look forward to future, noninvasive technological advances that will reinterpret the present human data (Figure 2 and Figure 3, Table 3) as hemispheric waves of activations that traverse the brain in milliseconds [27].

## 5. Conclusions

The complex, dynamic interactions of subconscious regulatory responses with conscious perceptions were observed during three experimental phases. First, during a 24-h water restriction period while conducting daily activities on Day 1, VAS ratings of thirst, desire for water, and mouth dryness paralleled the increase of plasma osmolality and dehydration. Thirst initially appeared at a mean body mass loss of 0.64 kg (0.8%). Second, during the 30-min seated laboratory oral rehydration session on the morning of Day 2, VAS ratings (thirst, desire for water, mouth dryness, stomach emptiness) rapidly decreased to their lowest points, in response to a mean 1.41 L voluntary fluid intake. No hydration index or VAS rating predicted the rehydration volume. Third, during ad libidum rehydration while conducting daily activities (Day 2), the patterns of change for plasma osmolality and VAS ratings differed and their synchrony decreased, unlike during water restriction on Day 1. At the end of this 24-h rehydration period (morning of Day 3), conscious perceptions (i.e., mouth dryness and stomach emptiness) predominated over osmotic signals (i.e., plasma osmolality) after test subjects had regained a euhydrated state. These findings represent unique interactions of subconscious responses with perceptions at each of the three experimental phases.

## Figures and Tables

**Table 1:** Comparison of ad libitum dietary content of Day 1 during which no fluids and only dry food items were consumed versus Day 2 during which fluids were consumed ad libitum.

Dietary Components	24-h Water Restriction (Day 1)	24-h Ad libitum Rehydration (Day 2)
Food energy content (Kcal)	2,219 ± 897	2377 ± 989
Sodium (mg)	4377 ± 2446	3755 ± 2090
Carbohydrate (%) ^a^	42 ± 12	46 ± 10
Fat (%) ^a^	39 ± 10	33 ± 7
Protein (%) ^a^	20 ± 5	21 ± 9
Food moisture + water + beverages (L) ^b^	0.31 ± 0.24	1.13 ± 0.52 ^c^
REHY water (L) ^d^	0 ± 0	1.46 ± 0.47
Total daily water intake (L)	0.31 ± 0.24	2.60 ± 0.66 ^c^

^a^, expressed as % of total caloric intake; ^b^, food diaries were analyzed using commercial dietary analysis software; ^c^, significantly different from Day 1 (row 6, *p* = 0.0002; row 8, *p* = 3.2 × 10^−8^); ^d^, measured by an investigator after a 30-min ad libitum rehydration period (REHY).

**Table 2:** Hydration-relevant variables, measured at the end of each 24-h phase, indicate that test subjects complied with the 24-h experimental intervention of water restriction and consumption of dry food items.

Measured Variables	24-h Water Restriction (Day 1 Morning to Day 2 Morning)	24-h Ad libitum Rehydration (Day 2 Morning to Day 3 Morning)
Body Mass change (kg/24 h)	−1.67 ± 0.35	+1.19 ± 0.36 ^a^
Body Mass change (%/24 h)	−2.12 ± 0.50	+1.56 ± 0.60 ^a^
Plasma Osmolality (mOsm/kg) ^b^	299 ± 5	294 ± 4 ^a^
Urine specific gravity ^b^	1.030 ± 0.003	1.021 ± 0.008 ^a^
Urine specific gravity ^c^	1.025 ± 0.005	1.017 ± 0.008 ^a^
Urine Osmolality (mOsm/kg) ^c^	893 ± 178	613 ± 279 ^a^
Urine Volume (L/24 h) ^c^	0.810 ± 0.275	1.770 ± 1.136 ^a^

^a^, significantly different from 24-h water restriction (range, *p* = 0.003 to *p* = 1.7 × 10^−10^); ^b^, single sample collected on the second morning of each observation period; ^c^, 24 h urine collection.

**Table 3:** Mean (±SD) values recorded at baseline and when test participants became aware of thirst during daily activities and returned to the laboratory.

Measurements	Baseline (Day 1, 0700 h)	Thirst Awareness ^a^ (Day 1)
Body mass (kg)	80.11 ± 10.58	79.47 ± 10.55
Plasma osmolality (mOsm/kg) ^b^	297 ± 4	298 ± 4
Urine specific gravity ^b^	1.020 ± 0.007	1.024 ± 0.004 ^c^
Thirst ^d^	55 ± 14	79 ± 12 ^c^
Desire for water ^d^	55 ± 18	79 ± 14 ^c^
Mouth dryness ^d^	58 ± 15	71 ± 22 ^c^
Stomach emptiness ^d^	57 ± 15	48 ± 28

^a^, this visit to the HPL occurred at a different time for each subject and represented a mean body mass loss of 0.64 kg (0.8%); ^b^, single sample; ^c^, significantly different from Baseline (range, *p* = 0.043 to *p* = 1.8 × 10^−5^); ^d^, Visual Analog Scale rating (0–100 range).
